# Translation and validation of the Chinese version of the MD Anderson symptom inventory for measuring perioperative symptom burden in patients with gynecologic cancer

**DOI:** 10.1186/s12905-021-01415-0

**Published:** 2021-07-29

**Authors:** Ting Zhang, Ying-ying Zheng, Zhi-rong Yang, Qiuling Shi, Xin Shelley Wang, Jun Zhao, Min Yang, Chun-lin Wu, Guo-rong Wang

**Affiliations:** 1 Gynecological Oncology Department of Sichuan Cancer Hospital and Institute, Sichuan Cancer Center, School of Medicine, University of Electronic Science and Technology of China, Chengdu, China; 2 School of Nursing, Chengdu University of Traditional Chinese Medicine, Chengdu, China; 3 Nursing Study Center, Sichuan Cancer Hospital and Institute, Sichuan Cancer Center, School of Medicine, University of Electronic Science and Technology of China, No. 55, 4Th Section of Renmin South Road, Chengdu, Sichuan China; 4 School of Public Health and Management, Chongqing Medical University, Chongqing, China; 5 Hospital Infection Department of Sichuan Cancer Hospital and Institute, Sichuan Cancer Center, School of Medicine, University of Electronic Science and Technology of China, Chengdu, China; 6 Department of Symptom Research, The University of Texas MD Anderson Cancer Center, Houston, TX USA

**Keywords:** Gynecology, Neoplasms, Symptom assessment, Chinese

## Abstract

**Background:**

Gynecologic cancers are among the most prevalent malignancies in China. Cervical and uterine cancer respectively account for the sixth and eighth highest incidence of cancer among Chinese women. Abdominal surgery is one of the important treatment methods for gynecological tumors. However, the tumor- and surgery-related symptom burden are not well studied owing to a lack of a standardized and validated assessment tool in the Chinese population. The study aimed to translate and validate the MD Anderson Symptom Inventory for measuring perioperative symptom burden in gynecologic cancer patients (MDASI-PeriOp-GYN) and examine the utility of the Chinese version of MDASI-PeriOp-GYN.

**Methods:**

The MDASI-PeriOp-GYN was translated in a stepwise manner. First, two native speakers independently translated the 9 PeriOp-GYN symptom items. Then the nine items were translated back into English by two different bilingual translators. After discussion and revision, the four translators reached an agreement. Finally, the finalized Chinese version was administered to women with three common gynecologic cancer types (cervical, ovarian, and endometrial cancers) recruited from the gynecological oncology department of Sichuan Cancer Hospital & Institute between July and October 2019. The reliability and validity of the translated version were assessed.

**Results:**

Overall, 324 women with gynecologic cancers were enrolled. Cronbach’s α values were 0.826 and 0.735 for the symptom severity and interference scales, respectively. Test–retest reliability values were 0.885, 0.873, and 0.914 for symptom severity, PeriOp-GYN, and interference scales. Significant correlations were found between the MDASI-PeriOp-GYN-C and EORTC QLQ-C30 along with the QLQ-OV28 module (− 0.608–0.871, *P* < 0.001). Known-group validity was supported by significant differences in the scores of the four scales grouped by time intervals, surgery type, and functional status (all *P* < 0.01).

**Conclusions:**

The MDASI-PeriOp-GYN-C is a valid and reliable tool for measuring symptoms in Chinese patients undergoing surgery for gynecologic cancers. The tool could be used in clinical practice and clinical trials to instantly gather patients’ health and quality of life data.

**Supplementary Information:**

The online version contains supplementary material available at 10.1186/s12905-021-01415-0.

## Key Message

- The Chinese version of the MDASI-PeriOp-GYN has shown excellent cross-cultural validity and reliability.
- The Chinese version of the MDASI-PeriOp-GYN could be used for measuring symptom severity and related interference with functioning for perioperative patients with gynecologic cancers in China.

## Introduction

Gynecologic cancers, particularly cervical, ovarian, and endometrial cancer, rank among the top 10 causes of female morbidity and mortality in China [1]. Surgery is one of the main treatment options, particularly for early-stage gynecologic cancer patients. However, tumor- and surgery-related symptoms have negative effects on patients’ functional status and quality of life [2, 3]. Active management of the perioperative symptom burden can reduce or even prevent postoperative complications, prompt initiation of postoperative supplementary therapy, and avoid potential reduction in progression-free survival [4]. An important factor in identification of symptoms is using a standardized and validated assessment tool. Nonetheless, no tools adapted for Chinese patients are currently available for assessing the perioperative symptom burden of gynecologic cancers.

The MD Anderson Symptom Inventory (MDASI) is a reliable and validated instrument for measuring common cancer-related symptoms [5]. Recently, MDASI modules for specific patient populations have been developed and psychometrically validated [6, 7]. Because of the anatomy of the female reproductive system and surgical scope, patients with gynecological tumors have special perioperative symptoms that MDASI did not mention, such as urinary and menopausal symptoms. A module of the MDASI for measuring perioperative symptom burden in gynecologic cancer patients (MDASI-PeriOp-GYN) was developed for patients undergoing gynecologic surgery and was found to be a valid, reliable, and concise tool [8]. Therefore, this study aimed to translate and validate the MDASI-PeriOp-GYN and examine the utility of the Chinese version (MDASI-PeriOp-GYN-C) to assess perioperative symptom burden in Chinese gynecologic cancer patients.

## Methods

### Translation and cultural adaption of the MDASI-PeriOp-GYN

The MDASI-PeriOp-GYN comprises the MDASI items and nine PeriOp-GYN symptom items [8]. The MDASI items include 13 core items assessing symptom severity and six items assessing symptom-related functional interference [5]. The tool was validated in various cancers, such as ovarian cancer and prostate cancer [9, 10], in different languages, such as Amharic and German [11, 12], and showed good reliability and construct validity. The MDASI-C was validated by Wang et al. in 249 Chinese cancer patients and used directly, where the Cronbach alpha coefficient was 0.87 for symptom subscale and 0.90 for interference subscale [13].

After obtaining consent from the original author, two native Chinese speakers fluent in English independently translated the nine PeriOp-GYN symptom items into Chinese characters. One of the translators had a master’s degree in nursing and ensured the accuracy and integrity of the items from a clinical perspective. With a master’s degree in English, the other kept the items concise from an ordinary person’s perspective. After the first Chinese version was created, two other bilingual translators who had not seen the original items back-translated this version to English. The four translators modified “clouding of consciousness” to “confusion” and “fever” to “hot flashes” in Chinese. Therefore, each item was rendered in its most intelligible form and easier to understand in Chinese. Finally, the Chinese version of the nine PeriOp-GYN symptom item was tested on 20 randomly selected postoperative patients to determine if the instructions, items, and options were clear and easily understandable and if there were confusing or offensive words. All items were clear and understandable to an ordinary person; thus, the MDASI-PeriOp-GYN-C was finalized.

### Design

Using a longitudinal design, a convenience sample completed the questionnaires before surgery and at 1, 5, and 7 days after surgery. According to a previous study [14], there were two peaks of postoperative symptom burden, with the highest symptom burden one day after surgery. Comparison with baseline levels showed the extent to which the tool detected differences between groups. The second peak was on day 6. Symptom burden was measured at day 5 and 7 after surgery to find the degree to which the measurement was consistent over time.

### Participants

Patients were recruited between July and October 2019 from the Sichuan Cancer Hospital. The inclusion criteria were as follows: (a) age ≥ 18 years; (b) diagnosis of cervical, ovarian, or endometrial cancer; (c) awareness of cancer diagnosis; (d) scheduled surgery; and (e) ability to read and speak Mandarin. The exclusion criteria included (a) major psychiatric illness; (b) primary malignant tumor of another site; (c) serious medical complications; (d) participation in other clinical trials that could affect this study, including trials of drugs for controlling symptoms.

### Procedures

Patients were recruited on hospital admission. All patients who met the inclusion criteria were invited to participate. The investigators explained that the survey was conducted at several time points, and written informed consent to participate was obtained. Patients were given the questionnaire before surgery and retrieved it after completion. After surgery, patients were distributed questionnaires at scheduled time points (discharged patients completed online surveys) so that test–retest correlations could be calculated.

The study was approved by the Ethics Committee of Sichuan Cancer Hospital (IIT2019018). All efforts were made to protect patients’ privacy and maintain data confidentiality.

### Measurements

For descriptive purposes, we evaluated the following socio-demographic and disease characteristics using a self-developed questionnaire: age, ethnicity, education, marital status, employment, chronic disease, tumor site, metastatic disease, cancer diagnosis, tumor metastasis, prior treatment, and type of surgery. The patient’s functional status was assessed using the Eastern Cooperative Oncology Group Performance Status (ECOG PS) Scale [1].

The MDASI-PeriOp-GYN was developed by Wang et al. in 2019. It contains 28 items grouped into two subscales (symptom and interference). Each item is scored from 0 (“not present” or “does not interfere”) to 10 (“the worst possible” or “interferes completely”). A score is reported for each item, as well as an overall score for each subscale. The higher the score, the greater the symptom burden. The original version has an internal consistency (Cronbach's alpha coefficient) of 0.86–0.89 [8].

The European Organization for Research and Treatment of Cancer quality of life questionnaire-core 30 (EORTC QLQ-C30) is a cross-culturally accepted cancer-specific health-related quality of life (HRQL) questionnaire that comprises five functioning scales: three symptom scales, six single-item scales, and a global quality of life scale [16]. The ovarian cancer module (OV28) of the EORTC QLQ contains disease-specific items related to the quality of life of ovarian cancer patients and was developed in a multicultural setting [17, 18]. The EORTC QLQ-OV28 comprises 28 items and is scored according to the EORTC conventions. A higher score represents higher symptom/problem levels. Both questionnaires should be used together. The Chinese version has been translated and validated by Chie et al. and had internal consistency reliability of 0.74–0.89 [19].

### Statistical analysis

IBM SPSS Statistics 22.0 (IBM, Armonk, NY, USA) was used for statistical analysis. All reported *P* values are 2-tailed. Statistical significance was set at *P* < 0.05. Following the method used in the original MDASI-PeriOp-GYN validation study, four scales were presented: MDASI-core (13 core items), PeriOp-GYN (nine items), symptom severity (22 items), and interference (6 items). The MDASI-PeriOp-GYN-C was examined for reliability and validity.

Reliability was evaluated based on internal consistency and test–retest reliability. The internal consistency was assessed using separate Cronbach coefficient α values for the four scales. For stability reliability, we established test–retest correlations by intra-class correlation (ICC) of data from two-time points (day 5 and 7 after surgery) because the patients’ condition was stable on these two days [14].

Methods for estimating validity included criterion validity and known-group validation. Criterion validity was examined by Spearman's rank correlation coefficient, and EORTC QLQ-C30 along with QLQ-OV28 was used as an external criterion. Known-group validation was examined by comparing the scores of the four scales between different time intervals, surgery type, and functional status. Independent two-sample t-tests and analysis of variance were used to compare the means between groups. When normality assumption was not satisfied, the nonparametric tests (e.g., Kolmogorov–Smirnov and Kruskal–Wallis tests) were used.

Exploratory factor analysis (EFA), including factor extraction and factor rotation, was applied to the measurement. Principal component analysis (PCA) was the approach to factor extraction. First, the Keiser-Meyer-Olkin (KMO) test for sampling adequacy and Bartlett’s test for sphericity were done to ensure that the EFA was adequate for PCA. Scree plot, eigenvalue, and component matrix followed. Each component explained the total variance. Component with eigenvalues greater than 1 were selected. After factor extraction, we interpreted the factor loadings using varimax orthogonal rotation; the goal was to improve the interpretability of the factor solution by reaching a simple structure.

## Results

### Response rate and patients characteristics

**Fig. 1:**
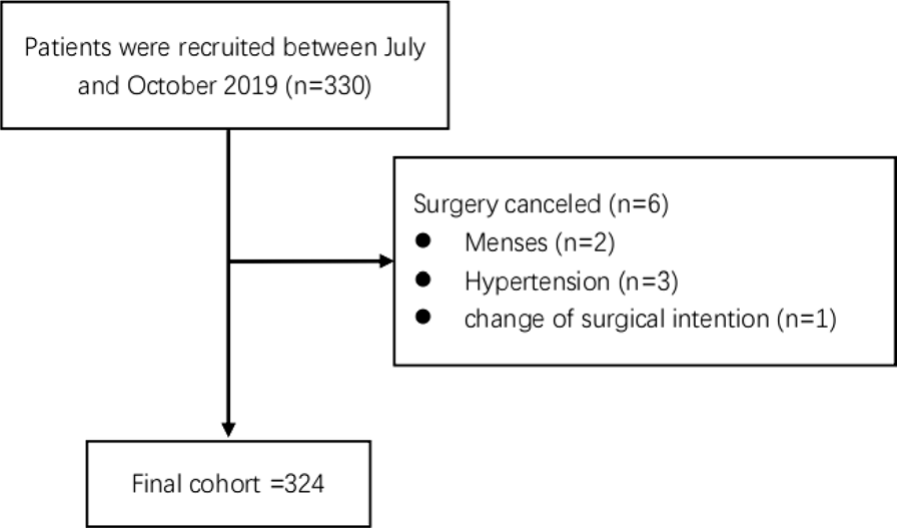
Flowchart of patients enrolled in the study

The effective response rate was 98.2% (324/330) (Fig. 1). Table 1 shows the demographic and disease-related patients’ characteristics. The patients’ age ranged from 22 to 75 years. The proportion of cervical, ovarian, and endometrial cancer was 46%, 31.2%, and 22.8%, respectively. Most patients (71.4%) underwent open surgery.

**Table 1:** Demographic and disease characteristics (n = 324)

Patient characteristics	n (%)
Age	
Mean ± SD (years)	51.95 ± 9.79
Ethnicity	
Han	316 (97.5)
Minority	8 (2.5)
Education	
Below high school	192 (59.3)
High school	105 (32.4)
College and above	27 (8.3)
Marital status	
Married	301 (92.9)
Single	7(2.2)
Divorced	7(2.2)
Widow	9(2.8)
Employment	
Employed	114 (35.2)
Retired	72 (22.2)
Others	138 (42.6)
Chronic disease	
Yes	31 (9.6)
No	293 (90.4)
BMI	
≤ 18.4	13(4.0)
18.5–23.9	164(50.6)
24–27.9	116(35.8)
≥ 28	31(9.6)
Cancer diagnosis	
Ovarian	101 (31.2)
Endometrial	74 (22.8)
Cervical	149 (46.0)
Stage	
I	203(62.8)
II	69(21.3)
III	43(13.3)
IV	9(2.7)
Prior treatment	
No treatment	210 (64.8)
Surgery	35 (10.8)
Chemotherapy	73 (22.5)
Radiotherapy	1 (0.3)
Surgery and chemotherapy	4(1.2)
Surgery, chemotherapy, radiotherapy	1 (0.3)
Type of surgery	
Open	232 (71.4)
MIS	92 (28.6)
ECOG PS	
0	251 (77.5%)
1	61 (18.8%)
2	12 (3.7%)

SD, standard deviation; MIS, minimally invasive surgery; ECOG PS, Eastern Cooperative Oncology Group Performance Status

### Reliability

The results for reliability are presented in Table 2. Cronbach’s α ranged from 0.826 for the symptom severity scale to 0.735 for the interference scale. Cronbach’s α was recomputed when each item was deleted, and the value raised to 0.74 when activity was deleted, indicating that activity could influence a low value of the interference scale.

**Table 2:** Internal consistency reliability for the MDASI-PeriOp-GYN-C

Item	Cronbach’s α	Cronbach’s α if item deleted
Symptom severity (22)	0.826	
MDASI-core items (13)	0.740	
Pain		0.816
Fatigue		0.812
Nausea		0.814
Sleeping disturbance		0.824
Distress		0.812
Shortness of breath		0.818
Memory		0.826
Poor appetite		0.806
Drowsiness		0.829
Dry mouth		0.824
Sadness		0.814
Vomiting		0.823
Numbness/tingling		0.826
Periop-GYN items (9)	0.821	
Bloating		0.810
Abdominal cramping		0.828
Constipation		0.823
Diarrhea		0.826
Dizziness		0.829
Grogginess/confusion		0.825
Urinary urgency		0.811
Inability to urinate/difficulty urinating		0.821
Hot flashes		0.824
Interference (6)	0.735	
Activity		0.740
Mood		0.733
Work		0.725
Relations		0.712
Walking		0.708
Enjoyment of life		0.723

MDASI-PeriOp-GYN-C, Chinese version of the MD Anderson Symptom Inventory for measuring perioperative symptom burden in patients with gynecologic cancer

The ICC was 0.922 for all items, 0.885 for the symptom severity scale, 0.873 for the PeriOp-GYN scale, 0.928 for the MDASI-core scale, and 0.914 for the interference scale (all *P* < 0.001).

### Validity

To assess the criterion validity, we examined the correlations between the MDASI-PeriOp-GYN-C and EORTC QLQ-C30 along with QLQ-OV28 (EORTC QLQ-OV58-C). Significant correlations were found for the symptom severity scale vs. the EORTC QLQ-OV58-C symptom scale (*r* = 0.871, *P* < 0.001), the MDASI-core scale vs. the EORTC QLQ-C30 symptom scale (*r* = 0.795, *P* < 0.001), the PeriOp-GYN scale vs. QLQ-OV28 symptom scale (*r* = 0.750, *P* < 0.001), and the interference scale vs. the EORTC QLQ-OV58-C functioning scale (*r* = − 0.608, *P* < 0.001).

The KMO score for the MDASI-PeriOp-GYN-C was 0.754, and Bartlett’s test for sphericity was significant (P < 0.001), indicating that data was suited for factor analysis. Under extraction, we selected seven factors that had eigenvalues greater than 1. The total variance explained by all factors was 74.85% (Table 3). Six symptoms (bloating, urinary urgency, pain, abdominal cramping, fatigue, inability to urinate/difficulty urinating) were sickness symptoms. Sleeping disturbance, distress, and sadness were emotional symptoms. Hot flashes and shortness of breath were endocrine problems. Dizziness, grogginess/confusion, drowsiness, and remember were cognitive symptoms. Vomiting, nausea, and poor appetite were upper gastrointestinal symptoms. Diarrhea and constipation were lower gastrointestinal symptoms. Dry mouth and numbness/tingling were classified within anesthetic symptoms.

**Table 3:** Factor loading, eigenvalues, and percent of variance for the MDASI-PeriOp-GYN-C items emerging from the principal component analysis (N = 324)

Item	Factor 1	Factor 2	Factor 3	Factor 4	Factor 5	Factor 6	Factor 7
Bloating	0.776	−	−	−	−	−	−
Urinary urgency	0.736	−	−	−	−	−	−
Pain	0.736	−	−	−	−	−	−
Abdominal cramping	0.499	−	−	−	−	−	−
Fatigue	0.453	−	−	−	−	−	−
Inability to urinate/difficulty urinating	0.449	−	−	−	−	−	−
Sleeping disturbance	−	0.785	−	−	−	−	−
Distress	−	0.689	−	−	−	−	−
Sadness	−	0.647	−	−	−	−	−
Hot flashes	−	−	0.684	−	−	−	−
Shortness of breath	−	−	0.633	−	−	−	−
Dizziness	−	−	−	0.773	−	−	−
Grogginess/confusion	−	−	−	0.753	−	−	−
Drowsiness	−	−	−	0.551	−	−	−
Remember	−	−	−	0.484	−	−	−
Vomiting	−	−	−	−	0.713	−	−
Nausea	−	−	−	−	0.639	−	−
Poor appetite	−	−	−	−	0.638	−	−
Diarrhea	−	−	−	−	−	0.861	−
Constipation	−	−	−	−	−	0.693	−
Dry mouth	−	−	−	−	−	−	0.745
Numbness/tingling	−	−	−	−	−	−	0.695
Eigenvalues	13.75	4.72	3.4	2.73	2.45	2.15	1.53
Total variance explained	33.50	11.5	8.27	6.65	5.96	5.23	3.73

Abbreviations: MDASI-PeriOp-GYN-C, Chinese version of the MD Anderson Symptom Inventory for measuring perioperative symptom burden in patients with gynecologic cancer

Table 4 shows the known-group validity. In comparing the scores of the four scales grouped by time intervals, surgery type, and functional status, the differences were statistically significant (*P* < 0.01; Table 4). On the first day after surgery, the scores for all scales were significantly higher than those before surgery (all *P* < 0.01). Patients undergoing open surgery reported higher scores in all scales than patients undergoing minimally invasive surgery (all *P* < 0.01).

**Table 4:** Known-group validity of the MDASI-PeriOp-GYN-C

Variable		n	Mean	SD	*t*	*P*
*Comparison by time*						
Symptom severity						
Preoperative		324	20.92	13.12	− 63.65	< 0.001
Postoperative day 1		324	62.61	12.29		
MDASI-core						
Preoperative		324	15.99	9.53	− 52.20	< 0.001
Postoperative day 1		324	43.10	9.63		
Periop-GYN						
Preoperative		324	4.93	4.86	− 45.44	< 0.001
Postoperative day 1		324	19.52	5.04		
Interference						
Preoperative		324	1.17	2.26	− 163.52	< 0.001
Postoperative day 1		324	25.24	2.87		
*Comparison by surgery type (postoperative day 1)*						
Symptom severity						
Open		232	66.36	10.71	− 9.94	< 0.001
MIS		92	53.17	10.89		
MDASI-core						
Open		232	45.84	8.81	− 9.11	< 0.001
MIS		92	36.18	8.83		
Periop-GYN						
Open		232	20.52	4.86	− 5.98	< 0.001
MIS		92	16.99	4.62		
Interference						
Open		232	25.87	2.71	− 6.61	< 0.001
MIS		92	23.67	2.64		
*Comparison by functional status (preoperative)*						
Symptom severity						
0–1		301	61.93	12.34	− 5.88	< 0.001
2–3		23	71.52	7.04		
MDASI-core						
0–1		301	42.63	9.68	− 3.19	0.002
2–3		23	49.17	6.47		
Periop-GYN						
0–1		301	19.30	5.09	− 2.82	0.005
2–3		23	22.35	3.2		
Interference						
0–1		301	25.11	2.890	− 4.45	< 0.001
2–3		23	26.96	1.821		

SD, standard deviation; MDASI-PeriOp-GYN-C, Chinese version of the MD Anderson Symptom Inventory for measuring perioperative symptom burden in patients with gynecologic cancer; MIS, minimally invasive surgery

### Clinical application of the MDASI-PeriOp-GYN-C

Table 5 presents the severity of all items across the survey period. On postoperative day 1, fatigue, drowsiness, and poor appetite were rated as the top three severe core symptom items (*P* < 0.05). Grogginess/confusion, hot flashes, and bloating were the most severe PeriOp-GYN items (*P* < 0.05). On postoperative days 5 and 7, fatigue and poor appetite were the most severe core symptom items, while bloating and hot flashes were the most severe PeriOp-GYN items (*P* < 0.05). After surgery, the interference scale scores decreased over time (all *P* < 0.001).

**Table 5:** Percentage and means of the MDASI-PeriOp-GYN-C scores according to time of survey

Item	Group 1: Pre-operation	Group 2: Postoperative day 1	Group 3: Postoperative day 5	Group 4: Postoperative day 7	*P*: All group comparison	*P:* Group 2 vs. 3 vs. 4
	Mean ± SD	Mean ± SD	Mean ± SD	Mean ± SD		
Symptom severity	20.97 ± 13.15	62.62 ± 12.29	22.67 ± 10.37	20.77 ± 8.07	< 0.001	< 0.001
MDASI-core items (13)	16.03 ± 9.55	43.11 ± 9.65	16.02 ± 6.99	14.85 ± 6.50	< 0.001	< 0.001
Pain	0.95 ± 1.24	4.11 ± 1.46	2.87 ± 0.78	2.24 ± 0.96		
Fatigue	3.13 ± 2.13	8.26 ± 1.34	4.26 ± 1.08	4.10 ± 1.10		
Nausea	0.68 ± 1.52	1.02 ± 1.37	0.13 ± 0.65	0.12 ± 0.51		
Sleeping disturbance	3.78 ± 2.03	5.39 ± 3.14	2.35 ± 1.96	2.50 ± 1.88		
Distress	1.71 ± 1.66	0.85 ± 1.35	0.80 ± 1.17	0.77 ± 1.05		
Shortness of breath	0.66 ± 1.25	1.28 ± 1.54	0.17 ± 0.66	0.14 ± 0.57		
Memory	0.37 ± 0.84	0.08 ± 0.60	0.03 ± 0.36	0.06 ± 0.34		
Poor appetite	2.32 ± 2.48	6.90 ± 2.91	3.11 ± 2.26	3.04 ± 2.21		
Drowsiness	0.43 ± 1.02	7.51 ± 1.76	0.68 ± 1.09	0.56 ± 0.97		
Dry mouth	0.30 ± 0.73	5.79 ± 1.86	0.27 ± 0.62	0.28 ± 0.57		
Sadness	1.09 ± 1.53	0.45 ± 0.93	0.67 ± 1.14	0.60 ± 1.10		
Vomiting	0.14 ± 0.64	0.08 ± 0.53	0.06 ± 0.57	0.02 ± 0.14		
Numbness/tingling	0.48 ± 0.95	1.38 ± 1.31	0.63 ± 0.93	0.56 ± 0.85		
Periop-GYN items (9)	4.93 ± 4.88	19.51 ± 5.03	6.65 ± 4.97	5.78 ± 2.85	< 0.001	< 0.001
Bloating	1.51 ± 1.93	3.44 ± 2.17	2.78 ± 1.69	2.58 ± 1.02		
Abdominal cramping	0.09 ± 0.45	0.48 ± 0.99	0.21 ± 1.07	0.09 ± 0.62		
Constipation	0.58 ± 1.22	0.04 ± 0.23	0.32 ± 1.27	0.12 ± 0.50		
Diarrhea	0.12 ± 0.62	0.44 ± 1.04	0.10 ± 0.63	0.08 ± 0.52		
Dizziness	0.70 ± 1.21	3.17 ± 1.60	0.99 ± 1.26	0.78 ± 0.09		
Grogginess/confusion	0.51 ± 1.04	7.43 ± 1.60	0.92 ± 1.24	0.76 ± 1.13		
Urinary urgency	0.96 ± 1.54	0.06 ± 0.42	0.07 ± 0.45	0.44 ± 0.94		
Inability to urinate	0.23 ± 0.91	0.13 ± 0.72	0.10 ± 0.63	0.04 ± 0.35		
Hot flashes	0.24 ± 0.86	4.33 ± 2.00	1.16 ± 1.62	0.91 ± 1.43		
Interference	1.24 ± 2.44	25.48 ± 3.59	18.02 ± 2.79	11.80 ± 3.69	< 0.001	< 0.001
Activity	0.09 ± 0.42	7.75 ± 1.30	4.45 ± 1.23	4.09 ± 1.66		
Mood	0.75 ± 1.35	0.45 ± 0.96	0.26 ± 0.72	0.31 ± 0.74		
Work	0.28 ± 0.75	9.99 ± 0.14	9.90 ± 0.52	4.39 ± 1.95		
Relations	0.06 ± 0.36	0.28 ± 1.53	0.08 ± 0.41	0.03 ± 0.24		
Walking	0.01 ± 0.08	6.86 ± 1.57	3.31 ± 1.19	2.96 ± 1.18		
Enjoyment of life	0.07 ± 0.33	6.86 ± 1.57	0.02 ± 0.18	0.03 ± 0.25		

SD, standard deviation; MDASI-PeriOp-GYN-C, Chinese version of the MD Anderson Symptom Inventory for measuring perioperative symptom burden in patients with gynecologic cancer

## Discussion

We have demonstrated the newly translated Chinese version of the MDASI-PeriOp-GYN to be a highly reliable and valid instrument for measuring symptom severity and related interference in perioperative Chinese gynecologic cancer patients, even with frequent measurement. Furthermore, integration of the numeric rating scale into the hospital information system or other computer systems or use as an online questionnaire makes this self-administered assessment to be convenient and timely, which may contribute to a better clinical outcome [20]. To our knowledge, this is the first study reporting reliability and validity data for the perioperative symptom burden in Chinese gynecologic cancer patients.

The mean age of our participants was 51.95 years, consistent with the results of epidemiological studies on gynecologic cancers in China. The age-specific incidence of cervical cancer increases rapidly from the 35–39 years age group, with the peak incidence in the 45–49 years age group [21, 22]. Ovarian cancer incidence increases after 40 years of age, reaching its peak in the 55–59 years age group [23]. Most of our participants (92.9%) had a spouse or domestic partner due to the traditional marriage and family in China.

Significantly, a high percentage of patients were free of chronic diseases. The incidence of hypertension and diabetes in Chinese adults was 27.9% and 10.9%, respectively; older adults, males, and urban residents had a higher prevalence [24]. However, only 36.5% of diabetes patients and 30.5% of hypertension patients had been diagnosed by doctors [25, 26]. Among the study participants, 74 (22.8%) patients were diagnosed with endometrial cancer that is closely related to hypertension and diabetes, which may be the reason for the small proportion of chronic disease patients among the participants.

Criterion validity was evaluated by comparing the responses on the MDASI-PeriOp-GYN-C with those on the Chinese version of the EORTC QLQ-C30 and its EORTC QLQ-OV28 module. The QLQ-C30 is the most widely used HRQL assessment in women with gynecologic cancers [27]. The use of QLQ-C30 and its QLQ-OV28 module could be recommended when the outcomes of interest are the core domains of HRQL or symptoms [28], while the MDASI-PeriOp-GYN-C focused on symptoms and interference with functioning. Cancer-related symptoms greatly influence the patients’ quality of life and might cause postoperative complications and delayed rehabilitation. Our study showed high correlations between the symptom and interference scales of the two questionnaires, with values > 0.6.

The results showed that symptom severity changed dramatically within seven days after surgery, consistent with the original study’s findings [8]. Professionals should conduct effective symptom management based on patient-reported outcomes to improve their quality of life and outcomes [29, 30]. Further studies are needed of symptom clusters in perioperative patients with different gynecologic cancers that affect the patients' quality of life to enable early treatment or prevention.

Within one week after surgery, fatigue was the most serious core symptom, followed by poor appetite, which was consistent with the results of previous studies [8, 31]. There are numerous causes of fatigue, including poor appetite, which might result in inadequate dietary intake, particularly energy and protein intake. Fatigue might also be due to insufficient activity after surgery [32]. Bloating and hot flashes were the most severe gynecologic symptoms. Hot flashes have been associated with hormone level changes after surgery while bloating is caused by impaired gastrointestinal function. In the Chinese culture, patience is a virtue and patients may be hesitant in expressing their discomfort. Therefore, the differences in symptom severity with patients in other countries should be explored.

The results showed that surgery had little effect on the patient's mood, relations, and enjoyment of life. With the effective control of pain, walking rapidly recovered within one week. However, the impact on activity and work was more persistent, which commonly including household activities and childcare. In the Chinese culture, women play an important role in these activities. Therefore, perioperative symptoms might continue to affect the daily life of patients and their families, and more social support should be offered to them. Multidisciplinary perioperative care, including rehabilitation medicine, is required for recovery and rehabilitation [33, 34].

This study had limitations. First, all participants were from the Chinese mainland and spoke Mandarin and simplified Chinese. Due to differences in the cultural background and mainstream languages in Hong Kong, Macao, and Taiwan, further study using the MDASI-PeriOp-GYN-C in these populations is needed. Second, 97.5% of the participants were of Han nationality. Differences in beliefs and living habits between the Han and minority nationalities may lead to differences in expressing perioperative symptoms, which need further evaluation. Third, this study only included patients with three common gynecological tumors. Therefore, the MDASI-PeriOp-GYN-C should be validated in patients with other gynecologic cancers and benign tumors.

In conclusion, the MDASI-PeriOp-GYN-C is a valid and reliable tool for measuring symptoms in Chinese patients undergoing surgery for gynecologic cancers. The tool could be used in clinical practice and clinical trials to instantly gather patients’ health and quality of life data.

## Supplementary Information

**Additional file 1.** Original data from 324 patients.

## Data Availability

All data generated or analyzed during this study are included in this published article (and its Additional file 1).
